# Age Differences in Psychological Antecedents and Behavioral Consequences of Stigmatization Associated with COVID-19 among Koreans

**DOI:** 10.3390/ijerph19148594

**Published:** 2022-07-14

**Authors:** Seonwoo Kang, Jungsuk Kang

**Affiliations:** Department of Psychology, Jeonbuk National University, Jeonju-si 54896, Korea; parrot@jbnu.ac.kr

**Keywords:** COVID-19, stigmatization, attribution theory, primary appraisal model, COVID-19 preventive behavior, COVID-19 testing intention, age difference

## Abstract

The first goal of this study is to develop a conceptual model of the causal relationship between psychological antecedents (internal attribution, anger, dangerousness, fear) of stigmatization, stigmatization (public stigma, anticipated stigma), and the behavioral consequences (compliance with COVID-19 prevention guidelines, COVID-19 testing intention) of stigmatization associated with COVID-19. The second goal of the study is to investigate the age differences in the conceptual model between younger and older adults unconfirmed with COVID-19 in Korea. After building the model based on previous studies, an online survey was conducted with Koreans in their 20s (*n* = 300, females: 50%) and 60s (*n* = 300, females: 50%) who had not been confirmed with COVID-19. The results revealed that for participants in their 20s and 60s, their internal attribution of COVID-19 infection to individuals confirmed with COVID-19 enhanced their anger at the individuals. Afterward, their anger increased their anticipated stigma of being confirmed with COVID-19 through enhancing the public stigma of the individuals confirmed with COVID-19. Unexpectedly, the fear of individuals confirmed with COVID-19 elicited by the dangerousness of the individuals had no effect on the public stigma of the individuals among participants in their 20s and 60s. The fear directly enhanced their compliance with the COVID-19 prevention guidelines. Next, for participants in their 20s, their anticipated stigma increased their compliance with COVID-19 prevention guidelines, but not their COVID-19 testing intention. However, the anticipated stigma did not affect both the compliance with the COVID-19 prevention guidelines and COVID-19 testing intention among participants in their 60s. The implications and limitations of these findings are discussed.

## 1. Introduction

As some infectious diseases including severe acute respiratory syndrome (SARS), Middle East respiratory syndrome (MERS), and Ebola virus disease are rapidly transmitted through personal contacts with infected individuals, stigmatization of the infected individuals with these diseases has occurred in their societies [1,2,3]. As a result, the stigmatization of the infected individuals during the outbreak of these infectious diseases has become a critical social issue. In the context of infectious disease outbreaks, stigmatization refers to a social process embedded in interpersonal relationships that devalues individuals who are confirmed with infectious diseases, inducing a high fatality rate [4]. COVID-19, as an infectious disease, has recently spread and caused a large number of deaths around the world [5]. The World Health Organization [6] has warned that the COVID-19 pandemic could provoke the stigmatization of individuals confirmed with COVID-19 (hereafter ICC) in their societies. 

Stigmatization associated with COVID-19 has negative effects on mental health at the individual and societal levels [7]. The stigmatization leads to social rejection and the avoidance of ICC [8]. At the individual level, the ICC may feel depressed and anxious due to the stigmatization [9,10]. Such negative emotions may last and reduce their social interactions for a long time after their recovery from COVID-19 [11,12]. Consequently, the stigmatization has harmful effects on the mental health among the ICC. At the societal level, a group of individuals unconfirmed with COVID-19 (hereafter IUCC) is more likely to feel fear of and angry at the other group of ICC. A group of IUCC (ingroup and majority) may tend to stigmatize and discriminate against the other group of ICC (outgroup and minority) [13,14]. This discrimination generates the IUCC’ discriminatory acts (e.g., verbal abuse, violent attacks) on the ICC [15]. Therefore, the stigmatization associated with COVID-19 thus undermines the overall mental health of societies. 

On the other hand, stigmatization related to COVID-19 negatively affects the physical health for IUCC [7]. Their concerns about being stigmatized by others resulting from their COVID-19 confirmation decrease their accessibility and acceptability of health care services associated with COVID-19 [16,17]. Such low accessibility and acceptability can be difficult to identify ICC among the IUCC, which may make the ICC miss their treatment timing, suffer from severe illnesses (e.g., pulmonary damage, pulmonary fibrosis), and die from COVID-19. Moreover, the anxiety of IUCC about being stigmatized in their societies due to their COVID-19 confirmation encourages them to conceal their COVID-19 symptoms and refuse their uptake of COVID-19 testing [18,19]. Their concealment of the symptoms and refusal of the testing may enhance the spread of COVID-19 and the mortality rate from COVID-19 in their societies. Consequently, the perception of IUCC of stigmatization has the potential to be injurious to not only their own physical health, but also to public physical health in their societies. 

To identify various barriers to the public’s positive responses to health care services and policies during the COVID-19 pandemic, it is critical to explore how stigmatization related to COVID-19 can influence adaptive (e.g., compliance with COVID-19 prevention guidelines) or maladaptive (e.g., refusal of COVID-19 testing) coping behaviors (behavioral consequences of the stigmatization) among the IUCC [19,20]. In addition, empirical investigation into the psychological antecedents of stigmatization among the IUCC has been limited [21]. The primary purpose of the study was thus to establish and verify a conceptual model of stigmatization associated with COVID-19 for IUCC in which its psychological antecedents and behavioral consequences are arranged in causal order. The conceptual model will integrate previous relevant theories or models. In addition, the study empirically examined age differences in the conceptual model. 

## 2. Conceptual Development

Past studies have suggested that attribution theory and the primary appraisal model can explain the psychological antecedents of stigmatization associated with COVID-19 [21,22,23,24]. The attribution theory and the primary appraisal model were integrated into a conceptual model of the stigmatization for IUCC in this study. The attribution theory and the primary appraisal model are discussed below in detail. 

According to attribution theory, the IUCC’ attribution (assignment of the cause) of the COVID-19 confirmation can influence their emotional responses to ICC [22,24]. The attribution consists of controllability, responsibility, and blame [25]. These components of attribution can be strongly associated with the IUCC’ stigmatization of the ICC [24]. When IUCC judge that the ICC can control their COVID-19 infection (controllability), the IUCC tend to assign the responsibility of contracting COVID-19 to the ICC. Consequently, IUCC are more likely to blame ICC. For the IUCC, their assigning controllability, responsibility, and blame of the COVID-19 infection to ICC may be called their internal attribution about COVID-19 infection [26]. Such an internal attribution makes IUCC feel angry at the ICC [22]. Therefore, the anger at ICC can evoke stigmatization of the ICC among the IUCC [22,24,27].

The primary appraisal model is a variation of the cognitive appraisal model [22]. The primary appraisal model suggests that the COVID-19 pandemic is an environmental stressor because it causes negative experiences such as lockdown, quarantine, infective sequelae, etc. [22,27,28]. Encountering the COVID-19 pandemic fortifies stress stemming from the risk of contracting COVID-19 among the IUCC [28]. For the IUCC, the stress is processed through primary appraisal [29]. Primary appraisal is a cognitive process through which IUCC evaluate whether the COVID-19 infection is dangerous [22,27,30]. When IUCC evaluate that the COVID-19 infection is dangerous, they are more likely to experience fear of encountering the ICC in their daily lives [27,29,30]. Fear of ICC can lead to the stigmatization of the ICC among the IUCC [22,27].

Stigma is defined as the occurrence of labeling, stereotyping, separation, social status loss, and the discrimination of individuals who possess a devalued attribute [31]. A variety of stigmas (e.g., self-stigma, perceived stigma) can play a role in developing stigmatization in societies [4,31]. Among these stigmas, public stigma [32] and anticipated stigma [19] tend to evoke the IUCC’ stigmatization of the ICC during the COVID-19 pandemic. In regard to the stigmatization associated with COVID-19, public stigma and anticipated stigma can be defined as follows. First, public stigma is the perceived occurrence of labeling, stereotyping, separation, social status loss, and the discrimination of the ICC among a majority of the IUCC [4,31]. Anticipated stigma is the IUCC’ expectation of experiencing labeling, stereotyping, separation, social status loss, and discrimination of them if others know that they are infected with COVID-19 [31,33,34].

Stigmatization associated with COVID-19 occurs due to public stigma and anticipated stigma [31]. For the IUCC, public stigma is more likely to facilitate the process of internalizing negative messages about the stigma (e.g., separation, discrimination) related to COVID-19 [35]. This process fortifies anticipated stigma [35,36]. Consequently, public stigma will enhance anticipated stigma among IUCC. Public stigma evokes stigmatization of ICC through increasing anticipated stigma among IUCC. 

To control the spread of COVID-19 and maintain their public physical health in society, IUCC should comply with the COVID-19 prevention guidelines and take COVID-19 tests immediately after being suspected of COVID-19 infection. For IUCC, their stigmatization of the ICC can influence their adaptive (e.g., compliance with COVID-19 prevention guidelines) and maladaptive (e.g., refusal of COVID-19 testing) coping behaviors related to their own and public physical health.

Governments have offered COVID-19 prevention guidelines to control the spread of COVID-19 around the world. COVID-19 prevention guidelines refer to a set of instructions on how individuals should behave to counteract COVID-19 infection (e.g., social distancing, washing hands, wearing face masks) [37,38]. The compliance with COVID-19 prevention guidelines can be directly increased by the fear of the ICC [39,40,41]. In addition, this fear may affect the compliance through influencing stigmatization associated with COVID-19. A majority of IUCC are well aware that their compliance with COVID-19 prevention guidelines will help reduce their infection of COVID-19. They are also anxious that their confirmation of COVID-19 can allow them to be stigmatized by others in their society. Their anxiety about being stigmatized due to the COVID-19 confirmation is more likely to encourage them to comply with the COVID-19 prevention guidelines [42,43]. Consequently, for the IUCC, an increase in their perception of stigmatization will lead to their compliance with the COVID-19 prevention guidelines. 

COVID-19 testing helps identify and quarantine individuals infected with COVID-19 and provides them with appropriate treatments. COVID-19 testing plays an important role in reducing the prevalence of COVID-19 and the mortality rate resulting from COVID-19 infection [19,44,45]. Most individuals infected with COVID-19 are asymptomatic or have mild clinical symptoms [46,47]. For such reasons, many infected individuals are not aware that they have contracted COVID-19. So, COVID-19 testing is the only way to identify who is infected with COVID-19. Many IUCC could be reluctant to take a COVID-19 test because they are concerned about being confirmed with COVID-19, which causes the stigmatization of the ICC [19,20]. For the IUCC, their perceived stigmatization of the ICC (anticipated stigma) will decrease their COVID-19 testing intention.

In conclusion, for the IUCC, their internal attribution of COVID-19 infection to the ICC will enhance their public stigma of the ICC through increasing their anger at the ICC. This pathway is suggested by attribution theory. In addition, for IUCC, the danger of the ICC will increase the public stigma through enhancing their fear of the ICC. This pathway is formulated based on the primary appraisal model. 

**Figure 1:**
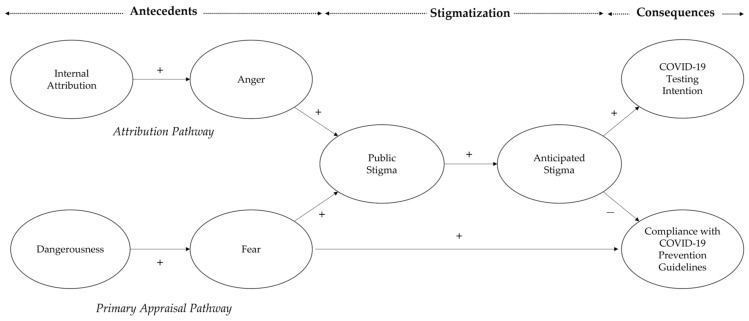
The conceptual model of the current study. Note: Positive sign (+) indicates a positive causal relationship between two variables whereas a negative sign (−) indicates a negative causal relationship between two variables.

Next, the IUCC’ public stigma of the ICC will fortify their anticipated stigma of being confirmed with COVID-19 in the future. Finally, the IUCC’ anticipated stigma will increase their compliance with the COVID-19 prevention guidelines and decrease their intention to undergo COVID-19 testing. Furthermore, the IUCC’ fear of the ICC can have a direct and positive effect on their compliance with the COVID-19 prevention guidelines. The causal relationship is depicted in Figure 1.

The relationship between the stigmatization associated with COVID-19, its antecedents, and its consequences (see the conceptual model in Figure 1) proposed by this study may differ between younger and older adult IUCC. For example, older adult IUCC are more vulnerable to COVID-19 infection because their immune functioning becomes weaker with age [48]. Compared to other age groups, they are at higher risk of suffering from severe illnesses (e.g., pulmonary damage, pulmonary fibrosis) and a higher mortality rate from COVID-19 infection [38,49,50,51]. Hence, older adult IUCC tend to regard COVID-19 infection as being more dangerous than younger adult IUCC [52,53]. Given that the dangerousness can positively affect fear of the ICC, the fear is more likely to increase public stigma than anger at the ICC for older adult IUCC. Compared to older adult IUCC, the anger may have a more positive effect on the public stigma than the fear among younger adult IUCC. Consequently, the primary appraisal (vs. attribution) pathway in the conceptual model is expected to affect the public stigma more strongly than the attribution (vs. primary appraisal) pathway for older (vs. younger) adult IUCC. 

Additionally, older adult IUCC may hold stronger public stigma associated with COVID-19 than younger adult IUCC [54]. This is because the cultural orientation of older adult IUCC is more collectivistic than the cultural orientation of younger adult IUCC [55]. The anticipated stigma fortified by the public stigma is more likely to affect compliance with the COVID-19 prevention guidelines and COVID-19 testing intention for older adult IUCC than for younger adult IUCC. However, it is difficult to predict how the causal relationship between stigmatization associated with COVID-19, its antecedents, and its consequences (see the conceptual model in Figure 1) may differ between younger and older adult IUCC. The study therefore explores the potential age-related differences in the conceptual model between them, based on the following research question. 

Research question: Will the causal relationship between stigmatization associated with COVID-19, its psychological antecedents, and its behavioral consequences differ between younger and older adult IUCC?

## 3. Methods

### 3.1. Participants

Korea is well-known as being a country to have controlled the outbreak of COVID-19 successfully [44,56]. Nevertheless, its mortality rates from COVID-19 infection for age groups were 55.5% (Koreans aged over 80 years), 28.2% (Koreans aged 70–79 years), 12.0% (Koreans aged 60–69 years) and 4.3% (Koreans aged under 59 years) on 5 January 2021 [57]. Hence, Koreans aged over 60 years perceived COVID-19 infection as being more severe than Koreans aged under 59 years. To reduce the selection bias related to the low Internet usage rate of potential study participants in an online survey for this study, Koreans aged 60–69 years were selected as the study population of older adults. This is because they had a higher Internet usage (91.5%) in comparison to Koreans aged over 70 years (40.3%) [58]. Koreans in their 20s were selected as the study population of younger adults. Based on sampling quotas by gender and age groups, Koreans aged 20–29 years and 60–69 years who had never had COVID-19 or taken a COVID-19 test were recruited from panel members of a Korean research company via email. A sample of 300 participants in their 20s (mean age = 26.12, *SD* = 2.35; females: 50%) and another sample of 300 participants in their 60s (mean age = 63.68, *SD* = 2.61; females: 50%) participated in the online survey. All participants consented to voluntarily participate in the online survey.

As the population density increases, the spread of COVID-19 increases [59]. The rapid spread of COVID-19 leads to an increase in the stigmatization associated with COVID-19 [7]. The spread of COVID-19 may be faster in metropolitan areas with a high population density than in non-metropolitan areas with a low population density. Stigmatization associated with COVID-19 can thus be stronger in metropolitan areas than in non-metropolitan areas. To explore the age differences in the conceptual model (see Figure 1) at the national level in Korea, participants living inside and outside metropolitan areas were recruited. There was no difference in the ratio of participants living in metropolitan areas (e.g., Seoul Metropolitan City, Incheon Metropolitan City) to participants living in non-metropolitan areas (e.g., Jeolla Province, Gangwon Province, Chungcheong Province) between the two age groups (20s vs. 60s) (χ^2^ (1) = 1.17, *p* > 0.05). 

### 3.2. Procedures and Measures

An online survey was conducted from 19 February to 29 February 2021. During this period, there was no large-scale spread of COVID-19 in Korea. In the online survey, participants reported their age (open-ended), gender (dichotomous), and residence (17 administrative areas in Korea). Next, they rated their collectivism on a 16-item (e.g., ‘It is my duty to take care of my family even when I have to sacrifice what I want.’), 7-point Likert-type scale adopted from a Korean study [60] (α = 0.73). Finally, the participants evaluated (a) the psychological antecedents (internal attribution, anger, dangerousness, fear) of stigmatization, (b) the stigmatization (public stigma, anticipated stigma), and (c) the behavioral consequences (compliance with COVID-19 prevention guidelines, COVID-19 testing intention) of stigmatization associated with COVID-19 on 7-point Likert-type scale. All 7-point Likert-type scales used in this study were anchored at the ends with the terms ‘1 = strongly disagree’ and ‘7 = strongly agree’. 

Reliability and validity tests for measures of the psychological antecedents, the stigmatization and the behavioral consequences were conducted after the data were collected from the online survey. As a result, the reliabilities of eight measures (α = 0.65–0.94) were acceptable [61]. Confirmatory factor analysis revealed that eight measures were valid (TLI = 0.80, CFI = 0.81, RMSEA = 0.06) [62,63]. The eight measures are discussed below in detail. 

#### 3.2.1. Psychological Antecedents of Stigmatization Associated with COVID-19

The internal attribution of COVID-19 infection to ICC was measured by 12 items (e.g., ‘Being confirmed with COVID-19 is a result of the person’s behavior.’) modified from the study by Mantler et al. [25] on a 7-point Likert-type scale (α = 0.80). Anger at ICC was gauged by three items (e.g., ‘I feel aggravated by a person confirmed with COVID-19.’) adapted from the study by Corrigan et al. [22] on a 7-point Likert-type scale (α = 0.91). The dangerousness of ICC was assessed by three items (e.g., ‘Persons confirmed with COVID-19 pose a risk to other people unless they are quarantined.’) modified from the study by Corrigan et al. [22] on a 7-point Likert-type scale (α = 0.84). Fear of ICC was measured by three items (e.g., ‘I feel scared by a person confirmed with COVID-19.’) adapted from the study by Corrigan et al. [22] on a 7-point Likert-type scale (α = 0.94). 

#### 3.2.2. Stigmatization Associated with COVID-19

The public stigma of ICC was assessed by 12 items (e.g., ‘Most young women would be reluctant to date a man who has been confirmed with COVID-19.’) modified from Link’s study [64] on a 7-point Likert-type scale (α = 0.84). Anticipated stigma of being confirmed with COVID-19 was measured by 10 items (e.g., ‘It would be socially unacceptable for me to be confirmed with COVID-19.’) adapted from the study by Rosenfield and Tomiyama [65] on a 7-point Likert-type scale (α = 0.92). 

#### 3.2.3. Behavioral Consequences of Stigmatization Associated with COVID-19

Compliance with COVID-19 prevention guidelines was gauged by 14 items (e.g., ‘At a restaurant, I keep wearing a face mask when I do not eat foods and consume drinks.’) developed from the Korean COVID-19 Prevention Guidelines [66] on a 7-point Likert-type scale (α = 0.84). COVID-19 testing intention was measured by three items (e.g., ‘I will get tested for COVID-19 after knowing that I visited a place where a person confirmed with COVID-19 had visited.’) developed from the Korean COVID-19 Prevention Guidelines [66] on a 7-point Likert-type scale (α = 0.65).

## 4. Results

The following statistical analyses were performed. First, an independent sample *t*-test was conducted to check the assumption that collectivism was stronger for participants in their 60s than for the participants in their 20s. Second, eight independent sample *t*-tests were performed to investigate differences in eight measures included in the conceptual model (see Figure 1) between two age groups (20s vs. 60s). Third, the correlation coefficients for 15 pairwise comparisons of the eight measures were calculated for each age group. Finally, structural equation modeling was used for each age group to test the age-related differences in the conceptual model.

### 4.1. Assumption Check

An independent sample *t*-test revealed that participants in their 60s (*M* = 4.25, *SD* = 0.84) showed a stronger collectivist tendency than participants in their 20s (*M* = 3.73, *SD* = 0.76) (*t*(*592.89*) = 7.87, variance equality was violated, *p* < 0.01). Hence, the results confirmed the assumption in this study.

### 4.2. Comparisons of Measures between Participants in Their 20s and 60s

Eight independent *t*-tests generated the following results. First, among the antecedents of stigmatization associated with COVID-19, there was no difference in their level of internal attribution between the two age groups (20s vs. 60s). However, anger, dangerousness, and fear were higher for participants in their 60s than for participants in their 20s. Second, participants in their 60s reported a higher level of public stigma than participants in their 20s, whereas participants in their 20s and 60s reported the same level of anticipated stigma. Finally, compliance with the COVID-19 prevention guidelines and COVID-19 testing intention were higher for participants in their 60s than for participants in their 20s (see Table 1 for details). 

### 4.3. Structural Equation Modeling for Participants in Their 20s and 60s

The correlation coefficients for the two age groups were calculated. The results are presented in Table 2. 

#### 4.3.1. Structural Equation Modeling for Participants Aged 20–29 Years

**Figure 2:**
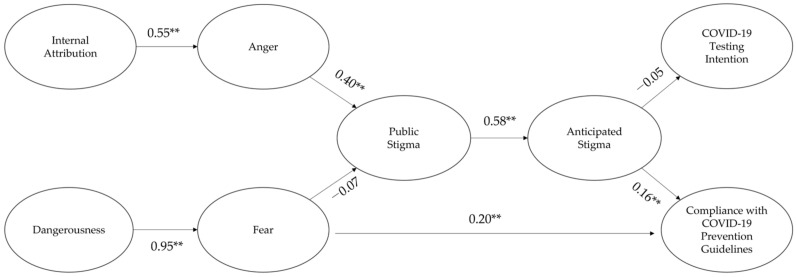
The standardized path coefficients for the conceptual model among participants in their 20s. ** *p* < 0.01.

For participants in their 20s, the structural equation modeling showed that the model fit was adequate (TLI = 0.80, CFI = 0.81, RMSEA = 0.06) [62,63]. The results are depicted in Figure 2 (see Table 3 for details). 

First, the internal attribution positively affected anger, which then enhanced the public stigma. Although dangerousness increased fear, fear had no effect on the public stigma. Second, fear had a direct and positive effect on compliance with the COVID-19 prevention guidelines. Third, the public stigma had a positive effect on the anticipated stigma. Finally, the anticipated stigma enhanced compliance with the COVID-19 prevention guidelines, but not the COVID-19 testing intention. 

#### 4.3.2. Structural Equation Modeling for Participants Aged 60–69 Years

**Figure 3:**
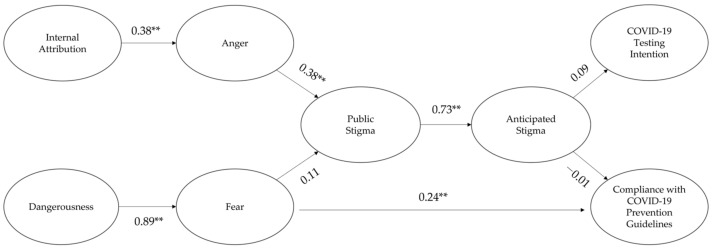
The standardized path coefficients for the conceptual model among participants in their 60s. ** *p* < 0.01.

For participants in their 60s, structural equation modeling showed that the model fit was adequate (TLI = 0.83, CFI = 0.843, RMSEA = 0.05) [62,63]. The results are depicted in Figure 3 (see Table 4 for details).

First, internal attribution enhanced the public stigma through increasing anger. Although dangerousness increased fear, fear had no effect on the public stigma. Second, fear had a direct and positive effect on compliance with the COVID-19 prevention guidelines. Third, the public stigma enhanced the anticipated stigma. Finally, the anticipated stigma had no effect on both the compliance with the COVID-19 prevention guidelines and the COVID-19 testing intention.

## 5. Discussion

The ultimate goal of this study is to investigate the age differences in a conceptual model of the causal relationship between the psychological antecedents (internal attribution, anger, dangerousness, fear) of stigmatization, stigmatization (public stigma, anticipated stigma), and behavioral consequences (compliance with COVID-19 prevention guidelines, COVID-19 testing intention) of stigmatization associated with COVID-19 between Korean younger and older adult IUCC (see Figure 2 and Figure 3). The study findings showed that for participants in their 20s and 60s who had not been confirmed with COVID-19, their internal attribution about COVID-19 infection to ICC enhanced their anger at the ICC, which then increased their public stigma of the ICC. Afterward, the public stigma had a positive effect on their anticipated stigma of being confirmed with COVID-19. However, their fear of ICC elicited by the dangerousness of the ICC did not affect the public stigma. These results support the attribution theory explaining the reason why the stigmatization associated with COVID-19 occurred [22,24], but not the primary appraisal model [22]. 

The study findings showed that fear of ICC directly enhanced their compliance with the COVID-19 prevention guidelines among participants in their 20s and 60s. However, the results revealed age differences in the relationship between their stigmatization and their compliance with the COVID-19 prevention guidelines. For participants in their 20s, their anticipated stigma fortified their compliance with the COVID-19 prevention guidelines, but not their COVID-19 testing intention. The anticipated stigma did not affect both the compliance with the COVID-19 prevention guidelines and the COVID-19 testing intention among participants in their 60s. Consequently, the results suggest that the fear of ICC can directly (fear → compliance with the COVID-19 prevention guidelines) and indirectly (fear → stigmatization → compliance with the COVID-19 prevention guidelines) affect the compliance with the COVID-19 prevention guidelines among younger adult IUCC. For older adult IUCC, their fear tends to have a direct and positive effect on their compliance without the mediation of their perceived stigmatization. 

The following unexpected results were found in this study. First, fear of ICC had no effect on the public stigma associated with COVID-19 among participants in their 20s and 60s who had not been confirmed with COVID-19. The following interpretation of the results is possible. For the IUCC, their fear of the ICC could generate their passive responses (e.g., freezing, withdrawal) to the ICC [67]. In contrast, their anger at the ICC may produce active responses (e.g., discriminatory thoughts, aggressive acts) toward the ICC [68]. The public stigma of the IUCC is strongly associated, not with the passive responses, but with the active responses to the ICC. As a result, the study findings showed that the fear was not related to the public stigma, whereas the anger was associated with the public stigma. 

Second, the anticipated stigma of being confirmed with COVID-19 did not affect the COVID-19 testing intention among the participants in their 20s and 60s who had not been confirmed with COVID-19. Unlike past studies [19,20] using measures for involuntary COVID-19 testing intention (e.g., ‘If a doctor ordered you to get tested for COVID-19, how likely are you to try to get tested?’), voluntary COVID-19 testing intention (e.g., ‘I will get tested for COVID-19 after knowing that I visited a place where a person confirmed with COVID-19 had visited.’) was measured in this study. This is because the Korea Disease Control and Prevention Agency encourages people to voluntarily get tested for COVID-19. Hence, the study findings offer the possibility that the voluntary COVID-19 testing intention could not be related to the anticipated stigma whereas the involuntary COVID-19 testing intention could be negatively associated with the anticipated stigma [20]. The results also suggest that IUCC may not perceive their voluntary COVID-19 testing as a method to magnify their anticipated stigma because they can freely decide whether they get tested for COVID-19 or not. 

Finally, for participants in their 60s, their anticipated stigma of being confirmed with COVID-19 did not influence their compliance with the COVID-19 prevention guidelines. The following interpretation of the results is possible. Participants in their 60s could regard COVID-19 confirmation as being more dangerous than participants in their 20s [52]. Their stronger fear of the ICC would directly affect their compliance with the COVID-19 prevention guidelines without increasing their perception of stigmatization (public stigma, anticipated stigma) associated with COVID-19 (see Figure 3) [69]. As a result, their perceived stigmatization (public stigma, anticipated stigma) did not mediate the effect of fear on the compliance with the COVID-19 prevention guidelines among older participants in this study.

In addition, the study explored age differences in the psychological antecedents of stigmatization, stigmatization, and the behavioral consequences of stigmatization associated with COVID-19 between Korean younger and older adult IUCC (see Table 2). The following results were obtained. First, the danger of ICC was higher for participants in their 60s than for participants in their 20s, which is consistent with the results of a past study [52].

Second, negative emotions (anger, fear) to ICC were more intensive among participants in their 60s than among participants in their 20s. The results can be interpreted as follows. Socioemotional selectivity theory suggests that to sustain positive emotions, older adult IUCC are less likely to attend to and memorize negative information associated with COVID-19 (e.g., increase in the mortality rate from COVID-19 infection) than younger adult IUCC [70,71]. Consequently, a negative relationship between age and negative emotional experiences was found during the COVID-19 pandemic [72]. However, according to the strength and vulnerability integration model, older adult IUCC have greater difficulties in effectively regulating emotions in their attention and memory than younger adult IUCC under conditions in which the COVID-19 pandemic is prolonged and inescapable [73]. Participants in their 60s thus experienced more intensive negative emotions (anger, fear) related to ICC than participants in their 20s. 

Third, the public stigma of the ICC was stronger for participants in their 60s than for participants in their 20s. The results are consistent with the results of a past study [54]. Stigmatization evoked by public stigma occurs more strongly among people holding collectivistic values than among people holding individualistic values [74]. Participants aged 60–69 years had a stronger collectivism tendency than participants aged 20–29 years in this study. Hence, participants in their 60s reported stronger public stigma than participants in their 20s. 

Finally, compliance with the COVID-19 prevention guidelines and COVID-19 testing intention were higher for participants in their 60s than for participants in their 20s. The findings can be due to the following reasons. The mortality rate from COVID-19 infection is higher among older adults than among younger adults in Korea [57]. Hence, the perceived severity of negative consequences (e.g., death, complications) generated from COVID-19 infection is higher among older adults than among younger adults. To avoid or limit the occurrence of such consequences, older adult IUCC (participants in their 60s) are more likely to engage in COVID-19 preventive behavior and COVID-19 testing than younger adult IUCC (participants in their 20s). 

To the authors’ knowledge, the current study is among the first to examine a conceptual model in which psychological antecedents of stigmatization, stigmatization, and the behavioral consequences of stigmatization associated with COVID-19 are causally related. The conceptual model integrates two theories (attribution theory, primary appraisal model) explaining how stigmatization associated with COVID-19 can occur. It was confirmed that the occurrence of the stigmatization associated with COVID-19 is well explained not by the primary appraisal model [22], but by attribution theory [22,24]. The study also showed that the impact of stigmatization on adaptive coping behavior (compliance with the COVID-19 prevention guidelines) differed between younger and older adult IUCC. 

When interpreting the study findings, some limitations should be considered. First, studies on stigmatization associated with COVID-19 can offer different results depending on the proliferation of COVID-19 (e.g., large-scale vs. small-scale spread of COVID-19) [20]. The online survey in this study was conducted when the small-scale spread of COVID-19 occurred in Korea. Hence, the study findings may not be generalized to a period when the number of ICC is exploding. To extend our understanding of the stigmatization associated with COVID-19, longitudinal studies to examine the relationship between the stigmatization, its psychological antecedents, and its behavioral consequences should be conducted as COVID-19 is spreading in Korea. 

Second, the study was performed with Koreans who hold stronger collectivism tendency than Westerners [75,76]. Given that there may be cultural differences (collectivist cultures vs. individualist cultures) in stigmatization, the study findings could not be generalized to Western countries. To verify the study findings across cultures, further studies with samples of Westerners are needed. 

Finally, the reliability of measure for the COVID-19 testing intention (α = 0.65) was lower than those of other measures, although the reliability was acceptable [61]. In addition, COVID-19 testing intention can be divided into two dimensions: voluntary and involuntary COVID-19 testing intention. For such reasons, future research should develop a valid and reliable scale to measure both the voluntary and involuntary COVID-19 testing intention. 

## 6. Conclusions

The study findings show that for Korean younger adult who had not been confirmed with COVID-19, the attribution pathway (internal attribution → anger → public stigma) can strengthen their COVID-19 protective behavior (compliance with the COVID-19 prevention guidelines) through fortifying their stigmatization associated with COVID-19, but not the primary appraisal pathway (dangerousness → fear → public stigma). However, the stigmatization does not mediate the effects of the attribution or primary appraisal pathway on the COVID-19 preventive behavior among Korean older adult who had not been confirmed with COVID-19. Consequently, the younger adults’ perceived stigmatization associated with COVID-19 can help reduce the spread of COVID-19 in Korea. This is because they are more likely to have face-to-face contact with many people frequently in their daily lives and are less likely to comply with the COVID-19 prevention guidelines. However, it is worth noting that the younger adults’ perceived stigmatization can harm the mental health of ICC because it may evoke discriminatory acts (e.g., verbal abuse, violent attacks) on the ICC. Their perceived stigmatization could also lead to social conflicts between them and the ICC in Korean society.

## Figures and Tables

**Table 1:** A comparison of the measures between the two age groups.

Factors	Measures	20s	60s	*t(df)*
Psychological antecedents	Internal attribution	3.99 ^1^ (0.73 ^2^)	4.01 (0.95)	0.24 (560.89 ^3^)
	Anger	3.80 (1.48)	4.47 (1.28)	5.97 (586.27) **
	Dangerousness	5.50 (1.15)	5.92 (0.87)	5.05 (555.79) **
	Fear	5.30 (1.33)	5.71 (1.02)	4.29 (560.64) **
Stigmatization	Public stigma	3.51 (0.86)	3.78 (0.85)	3.83 (598) **
	Anticipated stigma	3.88 (1.18)	3.98 (1.12)	1.06 (598)
Behavioral consequences	Compliance with COVID-19 prevention guidelines	4.87 (0.87)	5.27 (0.84)	5.82 (598) **
	COVID-19 testing intention	4.15 (1.34)	4.39 (1.41)	2.18 (596.13) *

^1^ Mean (*M*). ^2^ Standard Deviation (*SD*). ^3^ A number with two decimal places indicates that the variance equality was violated. * *p* < 0.05, ** *p* < 0.01.

**Table 2:** The correlation matrix for the two age groups.

Factors	1	2	3	4	5	6	7	8
1. Internal attribution	-							
2. Anger	0.39 **^,1^ (0.23 **^,2^)	-						
3. Dangerousness	0.11 (0.12 *)	0.42 ** (0.47 **)	-					
4. Fear	0.12 * (0.05)	0.50 ** (0.57 *)	0.87 ** (0.79 **)	-				
5. Public stigma	0.23 ** (0.22 **)	0.30 ** (0.38 **)	−0.03 (0.19 *)	0.03 (0.24 **)	-			
6. Anticipated stigma	0.29 ** (0.26 **)	0.43 ** (0.46 **)	0.24 ** (0.31 **)	0.30 ** (0.33 **)	0.47 ** (0.65 **)	-		
7. Compliance with the COVID-19 prevention guidelines	0.17 ** (0.11)	0.15 * (0.21 *)	0.23 ** (0.29 **)	0.24 ** (0.25 **)	0.08 (0.12 *)	0.15 * (−0.08)	-	
8. COVID-19 testing intention	0.06 (0.08)	−0.05 (0.07)	0.04 (0.05)	0.09 (0.01)	−0.01 (0.00)	−0.07 (0.08)	0.16 ** (0.11)	-

^1^ The number outside parenthesis indicates the correlation coefficient for participants in their 20s. ^2^ The number in parenthesis indicates the correlation coefficient for participants in their 60s. * *p* < 0.05, ** *p* < 0.01.

**Table 3:** The causal relationships between measures for participants in their 20s.

Factors	Path	b	Standardized b	SE ^1^	CR ^2^
Psychological antecedents	Internal attribution → Anger	2.15	0.56 **	0.62	3.49
	Anger → Public stigma	0.21	0.41 **	0.04	5.07
	Dangerousness → Fear	1.14	0.96 **	0.07	17.39
	Fear → Public stigma	−0.04	−0.08	0.03	−1.22
	Fear → Compliance with the COVID-19 prevention guidelines	0.09	0.20 **	0.03	2.65
Stigmatization	Public stigma → Anticipated stigma	0.85	0.59 **	0.13	6.38
Behavioral consequences	Anticipated stigma → Compliance with the COVID-19 prevention guidelines	0.09	0.17 *	0.04	2.26
	Anticipated stigma → COVID-19 testing intention	−0.07	−0.06	0.07	−1.02

^1^ Standard Error. ^2^ Critical Ratio. * *p* < 0.05, ** *p* < 0.01.

**Table 4:** The causal relationships between measures for participants in their 60s.

Factors	Path	b	Standardized b	SE ^1^	CR ^2^
Psychological antecedents	Internal attribution → Anger	0.39	0.38 **	0.07	5.80
	Anger → Public stigma	0.26	0.38 **	0.05	5.07
	Dangerousness → Fear	1.18	0.90 **	0.09	12.78
	Fear → Public stigma	0.09	0.12	0.05	1.84
	Fear → Compliance with the COVID-19 prevention guidelines	0.16	0.25 **	0.05	3.29
Stigmatization	Public stigma → Anticipated stigma	0.99	0.74 **	0.13	7.60
Behavioral consequences	Anticipated stigma → Compliance with the COVID-19 prevention guidelines	0.00	−0.01	0.04	−0.09
	Anticipated stigma → COVID-19 testing intention	0.16	0.10	0.11	1.44

^1^ Standard Error. ^2^ Critical Ratio. ** *p* < 0.01.

## Data Availability

Not applicable.
